# Arthroscopic Transplantation of Synovial Stem Cells Improves Clinical Outcomes in Knees With Cartilage Defects

**DOI:** 10.1007/s11999-015-4324-8

**Published:** 2015-04-30

**Authors:** Ichiro Sekiya, Takeshi Muneta, Masafumi Horie, Hideyuki Koga

**Affiliations:** Center for Stem Cell and Regenerative Medicine, Tokyo Medical and Dental University, 1-5-45 Yushima, Bunkyo-ku, Tokyo, 113-8519 Japan; Department of Joint Surgery and Sports Medicine, Graduate School, Tokyo Medical and Dental University, Tokyo, Japan

## Abstract

**Background:**

Transplantation of mesenchymal stem cells (MSCs) is one possible strategy to achieve articular cartilage repair. We previously reported that synovial MSCs were highly proliferative and able to undergo chondrogenesis. We also found that placing a suspension of synovial MSCs on a cartilage defect for 10 minutes promoted cartilage repair in rabbit and pig models. However, the in vivo efficacy of this approach has not been tested clinically.

**Questions/purposes:**

We asked whether transplantation of synovial MSCs improves (1) MRI features, (2) histologic features, and (3) clinical evaluation scores in patients with cartilage defects in the knee?

**Methods:**

Patients with a symptomatic single cartilage lesion of the femoral condyle were indicated for inclusion in our study, and between April 2008 and April 2011, 10 patients were enrolled in this study. All patients completed followups of 3 years or more. The average followup period was 52 months (range, 37–80 months). Synovial MSCs were expanded with 10% autologous human serum for 14 days after digestion. For transplantation, the patient was positioned so that the cartilage defect was facing upward, and synovial MSC suspension was placed on the cartilage defect with a syringe under arthroscopic control. The defect with the applied suspension then was held in the upward position for 10 minutes. Five patients underwent concomitant ACL reconstructions, among whom two had meniscus suturing performed simultaneously. For MRI quantification, the cartilage defect was scored from 0 to 5. Second-look arthroscopy was performed for four patients and biopsy specimens were evaluated histologically. Clinical outcome was assessed using the Lysholm score and Tegner Activity Level Scale at final followup. Comparisons of MRI and Lysholm scores before and after treatment for each patient were analyzed using the Wilcoxon signed-rank test.

**Results:**

MRI score (median ± 95% CI) was 1.0 ± 0.3 before and 5.0 ± 0.7 after, and increased after treatment in each patient (p = 0.005). Second-look arthroscopy in four patients showed that the cartilage defect appeared to be qualitatively better in all cases. Histologic analyses showed hyaline cartilage in three patients and fibrous cartilage in one at the deep zone. The Lysholm score (median ± 95% CI) was 76 ± 7 before and 95 ± 3 after, and increased after treatment in each patient (p = 0.005). The Tegner Activity Level Scale did not decrease after treatment in each patient.

**Conclusions:**

For this small initial case series, transplantation of synovial MSCs was effective in terms of MRI score, qualitative histology, and Lysholm score. The use of synovial MSCs has an advantage in that the cells can be prepared at passage 0 in only 14 days. Transplantation of synovial MSCs may be less invasive than mosaicplasty and autologous chondrocyte implantation. To conclusively show the effectiveness of this treatment requires comparative studies, especially with more established arthroscopic procedures, such as marrow stimulation techniques.

**Level of Evidence:**

Level IV, therapeutic study.

## Introduction

Articular cartilage injuries are a common clinical problem and if left untreated, may lead to osteoarthritis. Although there are various methods for surgical intervention, each has respective disadvantages: poor structural quality of the repaired cartilage in bone marrow stimulation, donor site morbidity in mosaicplasty, and loss of chondrogenic phenotype of expanded chondrocytes in autologous chondrocyte implantation [13]. Stem cell therapy may be one possible strategy for improving repair of cartilage injuries. One of the candidate therapeutic cells is mesenchymal stem cells (MSCs), which can be isolated from various mesenchymal tissues. Previous in vitro [21] and in vivo [8] chondrogenic assays showed that synovial MSCs had superior chondrogenic ability compared with MSCs from other tissues. Synovial MSCs also expanded well in the presence of human serum [19]. Finally, in rabbit [9] and pig [16] studies, transplantation of synovial MSCs promoted cartilage regeneration; therefore, synovial MSCs appear to be a promising cell source for cartilage repair.

The synovium is a thin membrane that covers the inside of the joint and has high regenerative potential [4]. According to previous studies, the number of MSCs in synovial fluid increased in knees with ACL injury [14], meniscus injury [12], and osteoarthritis [24]. The morphologic features and gene profiles of the MSCs released in synovial fluid after a joint injury were more similar to synovial MSCs than bone marrow MSCs. Principal component analysis of gene profiles for various mesenchymal tissue-derived MSCs and chondrocytes showed that MSCs from intraarticular tissues and chondrocytes were closer to each other than MSCs from extraarticular tissues [23]. Embryologically, chondrocytes and synovial cells share a similar progenitor-cell population [1]. Synovial MSCs which were injected intraarticularly attached to the injured site and promoted healing to various degrees in a rabbit cartilage-defect model [9]. The findings suggest that the synovium is a reservoir for MSCs that can contribute to intraarticular tissue repair. After intraarticular tissues like cartilage are injured, MSCs may be mobilized from the synovium to the synovial fluid, adhere to the injured site, and contribute to its repair. However, native MSCs are limited in quantity; this is likely the reason that injured articular cartilage generally does not heal. Transplantation of synovial MSCs in large numbers to injured tissues may promote a natural healing process for injured tissues including articular cartilage.

Various methods have been used to transplant MSCs in cartilage defects, such as intraarticular injection and transplantation, with or without the use of scaffolds [13]. It was shown that placing a suspension of synovial MSCs on the cartilage defect and leaving the cartilage defect immobilized for 10 minutes resulted in approximately 60% of the cells adhering to the defect to promote cartilage repair in rabbit [9, 25] and pig [16] knee models. The other 40% of synovial MSCs were taken up by adjacent synovial tissues, with no adverse effects on the synovium or other tissues in the knee. This technique for use of synovial cells can be performed arthroscopically without the need for synthetic or natural scaffolds. On the basis of more than 50 promising basic and preclinical research studies [3], we began arthroscopic transplantation of autologous synovial MSCs for cartilage defects in the knee.

We believe this is the first report of a clinical study performed with synovial MSCs; as such, the in vivo efficacy of this approach, to our knowledge, has not been tested. We asked whether transplantation of synovial MSCs improves (1) MRI features, (2) histologic features, and (3) clinical evaluation scores in patients with cartilage defects in the knee?

## Patients and Methods

Our study was approved by the ethics committee of our university. Eligible patients were 20 years old or older with a symptomatic single cartilage lesion of the femoral condyles (Table 1). Ten patients were enrolled in this study, and the cause of the cartilage defect primarily was trauma for all 10. The first patient was enrolled in April 2008 and the last in April 2011. All completed followups for 3 years or more. The median age of the patients was 41 years (range, 20–43 years); median duration of symptoms was 3 years (range, 0.6–16 years); median size of each cartilage defect was 200 mm^2^ (range, 25–500 mm^2^); and median followup was 48 months (range, 36–80 months). The inclusion criteria included “symptomatic International Cartilage repair Society (ICRS) Grades 3 and 4 cartilage single lesions of the femoral condyles” and the exclusion criteria included “less than 6 months with symptom”, “patellofemoral cartilage lesion”, and “microfracture performed” (Table 2). Five patients underwent ACL reconstructions, among whom two had meniscus sutures performed simultaneously with the synovial MSC transplantation.

**Table 1:** Details of 10 patients with femoral condyle defects treated with synovial MSCs

Patient	Age (years)	Sex	Duration of symptom (years)	Size of lesion (mm^2^)	Associated surgery	Previous surgery, characteristic features	Transplanted site	MRI evaluation (months)	Followup (months)	Transplanted cell number (×106)
1	26	M	2	225		ACL reconstruction, MM removal	MFC	3	63	43
2	43	M	3	500	Removal of free body	Osteochondral defect	LFC	72	80	7
3	26	M	9	162	ACL reconstruction	MM partial worn	MFC	12	47	34
4	21	M	4	54	ACL reconstruction	MM partial worn	MFC	12	37	77
5	42	F	16	500	ACL reconstruction	MM partial worn	MFC	6	56	39
6	20	F	1	25	ACL reconstruction, MM suture		MFC	3	65	72
7	41	F	3	200	ACL reconstruction, MM suture		MFC	3	49	40
8	40	F	0.6	120			MFC	24	44	50
9	41	M	8	400		LM removal	LFC	24	37	70
10	41	F	0.7	200			MFC	24	39	40

MSC = mesenchymal stem cell; MM = medial meniscus; LM = lateral meniscus; MFC = medial femoral condyle; LFC = lateral femoral condyle.

**Table 2:** Inclusion and exclusion criteria

Inclusion criteria
Patients provided written informed consent
20 years of age and older
Symptomatic cartilage single lesions of the femoral condyles
ICRS Grades 3 (cartilage defects extending down > 50% of cartilage depth) and 4 (bone defect)
Exclusion criteria
Less than 6 months with symptoms
Patellofemoral cartilage lesion
Microfracture performed
Pregnant female
Infectious diseases
Malignancy
Rheumatoid arthritis
Diabetes
Poor general health condition

ICRS = International Cartilage Repair Society.

**Fig. 1A–E:**
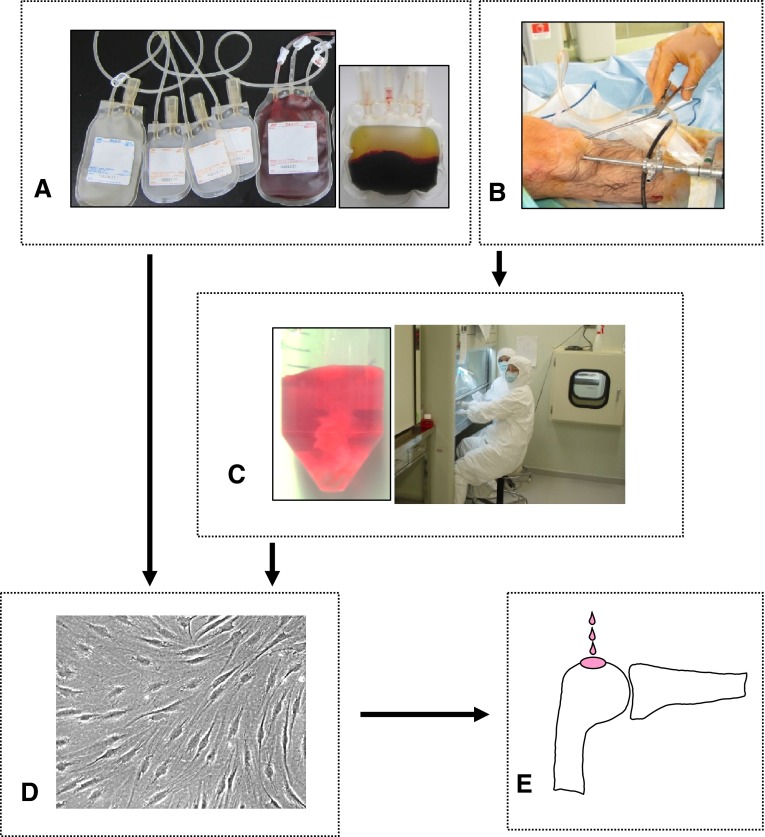
Preparation of synovial MSCs for arthroscopic transplantation is shown. (**A**) Peripheral blood was collected for autologous human serum. (**B**) Synovium was harvested with a pituitary rongeur under arthroscopic observation. (**C**) Synovium was digested at a cell processing center. (**D**) Synovial MSCs were expanded with 10% autologous human serum. (**E**) Passage 0 synovial MSCs were transplanted arthroscopically.

One or 2 days before synovial tissue was harvested, approximately 300 mL of whole blood was obtained from all donors using Cellaid^®^ (JMS Co Ltd, Hiroshima, Japan), a closed-bag system for isolation of serum (Fig. 1). The system consists of a blood donation bag containing glass beads which function by activating platelets and removing fibrin from whole blood through a 30-minute, gentle mixing process. After centrifugation at 2000 g for 7 minutes, the serum was isolated and heat inactivated at 56° C for 30 minutes. The serum was filtered through a 0.45-μm nylon filter (Becton Dickinson, Franklin Lakes, NJ, USA) and stored at 4° C until use [19].

Arthroscopy was performed to observe cartilage defects in Patients 1, 8, 9, and 10, who received local anesthesia with 20 mL 1% xylocaine. Then, with the patient under intravenous anesthesia with 0.1 g sodium pentothal, the synovium with subsynovial tissue on the femur at the suprapatellar pouch was harvested with a pituitary rongeur under arthroscopic observation (Fig. 1). Patients were discharged from the hospital after synovium harvest. For Patients 2 through 7 who received lumbar spinal anesthesia, the synovium with subsynovial tissue was harvested before ACL reconstruction, medial meniscus suture, or removal of free bodies was performed.

The cell culture was performed in the cell processing center at the authors’ institution. The cell processing center acquired ISO9001 certification, the international standard for quality management systems, in 2004. The synovium was digested in a solution of 5 mg Liberase™ (Roche Diagnostics, Mannheim, Germany) in 5 mL Hanks’ Balanced Salt Solution (HBSS; Invitrogen, Carlsbad, CA, USA) at 37° C (Fig. 1). After 3 hours, the digested cells were filtered through a 70-μm nylon filter (Becton Dickinson). The cells were cultured in α-MEM (Invitrogen), containing 10% autologous human serum, 100 units/mL penicillin, 100 μg/mL streptomycin, and 250 ng/mL amphotericin B. At 12 days, two among approximately 50 dishes were selected to examine bacteria, endotoxin in the medium, mycoplasma, and virus in the cells. For bacterial testing, chocolate agar was used. For endotoxin testing, a Toxicolor^®^ LS-50M kit (Seikagaku Corporation, Tokyo, Japan) was used [26]. For mycoplasma and virus tests, a multiplex PCR system developed by our team was used [6]. This system made it possible to detect 142 types of mycoplasma and 17 types of virus. One dish also was used for chromosomal testing.

After no contamination with bacteria, mycoplasma, virus, or endotoxin was confirmed, synovial MSCs were harvested at 14 days (Fig. 1D) with TrypLE™ (Invitrogen) at 37° C for 5 minutes. Thirty minutes before transplantation, primary synovial MSCs were suspended in 0.5 mL acetate Ringer’s solution (Veen-3G; Kowa, Tokyo, Japan) [25]. The number of transplanted cells was 47 ± 21 million (mean ± SD).

**Fig. 2A–C:**
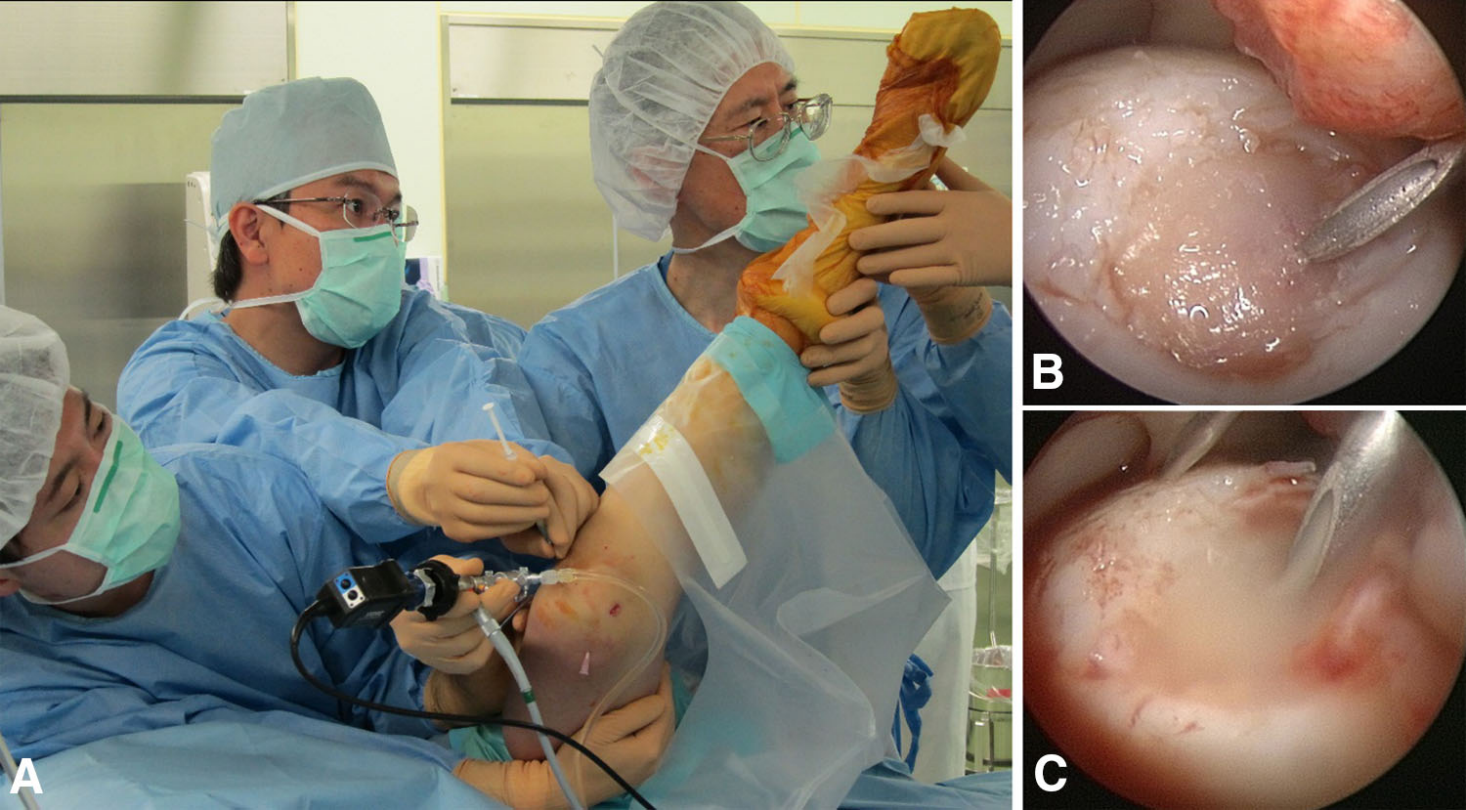
Arthroscopic transplantation of synovial MSCs is shown. (**A**) The patient was positioned so that the cartilage defect faced upward. This patient had a cartilage defect on the lateral femoral condyle. His hip was flexed, adducted, and internally rotated to face the cartilage defect upward. A suspension of synovial MSCs in 0.5 mL was placed in the defect through an 18-gauge needle attached to a 1-mL syringe. (**B**) Irrigation fluid was completely drained from the knee. (**C**) The synovial MSC suspension was placed on the cartilage defect of the femoral condyle. The patient was maintained in position for 10 minutes.

With the patient under lumber spinal anesthesia, the surface of the cartilage legion was arthroscopically scratched with curettage for débridement, but bleeding from the subchondral bone was avoided. The knee then was moved so the cartilage defect was facing upward (Figs. 1E, 2A), and irrigation fluid was completely drained from the knee (Fig. 2B). A suspension of synovial MSCs in 0.5 mL acetate Ringer’s solution was placed in the defect through an 18-gauge needle attached to a 1-mL syringe (Fig. 2C). The patient was maintained in position for 10 minutes [9, 25]. The incisions for the portals then were closed without washing the inside of the knee.

All patients started ROM exercise of the knee 1 day after the procedure, were partial weightbearing at 2 weeks, and full weightbearing at 6 weeks. Special equipment such as a continuous passive motion machine was not used. Generally, low-impact activities started at 3 months and high-impact activities were allowed at 6 months.

All MRI examinations were performed on a 3.0-T Gyroscan Intera MR unit (Philips Medical Systems, Best, The Netherlands). MR images were taken with 10° flexion of the knee. For quantification, grading for “degree of defect repair and filling of the defect” as described by Marlovits et al. [11] was modified as “cartilage defect” for the purposes of our study. This score was evaluated by two independent observers (MH, KO) in a blinded manner. Sagittal and coronal MR images were assessed preoperatively and 3 months postoperatively for each patient. MRI followup was available for four of the 10 patients at a minimum of 2 years (mean, 18 months; range, 3–72 months) (Table 1).

Second-look arthroscopy was done on four patients who reported having discomfort with two staples on the tibia for ACL reconstruction [15], with the procedure for removal of staples at 11 to 18 months after transplantation of synovial MSCs. After informed consent was obtained, a needle biopsy also was performed at the center of the repaired cartilage. Biopsy specimens of the fragment were fixed in 4% paraformaldehyde for 24 hours, embedded in paraffin, and cut into 5-μm sections. The specimens were stained with Safranin O and fast green and viewed with an Olympus^®^ MVX10 microscope (Olympus Corporation, Tokyo, Japan). Cartilage matrix was described qualitatively.

Clinical outcomes were assessed using the Lysholm score [10] and Tegner Activity Level Scale [27] at final followup.

Comparison of the MRI and Lysholm scores before and after treatment for each patient were analyzed using the Wilcoxon signed rank test. A p value less than 0.05 was considered statistically significant.

## Results

**Fig. 3A–G:**
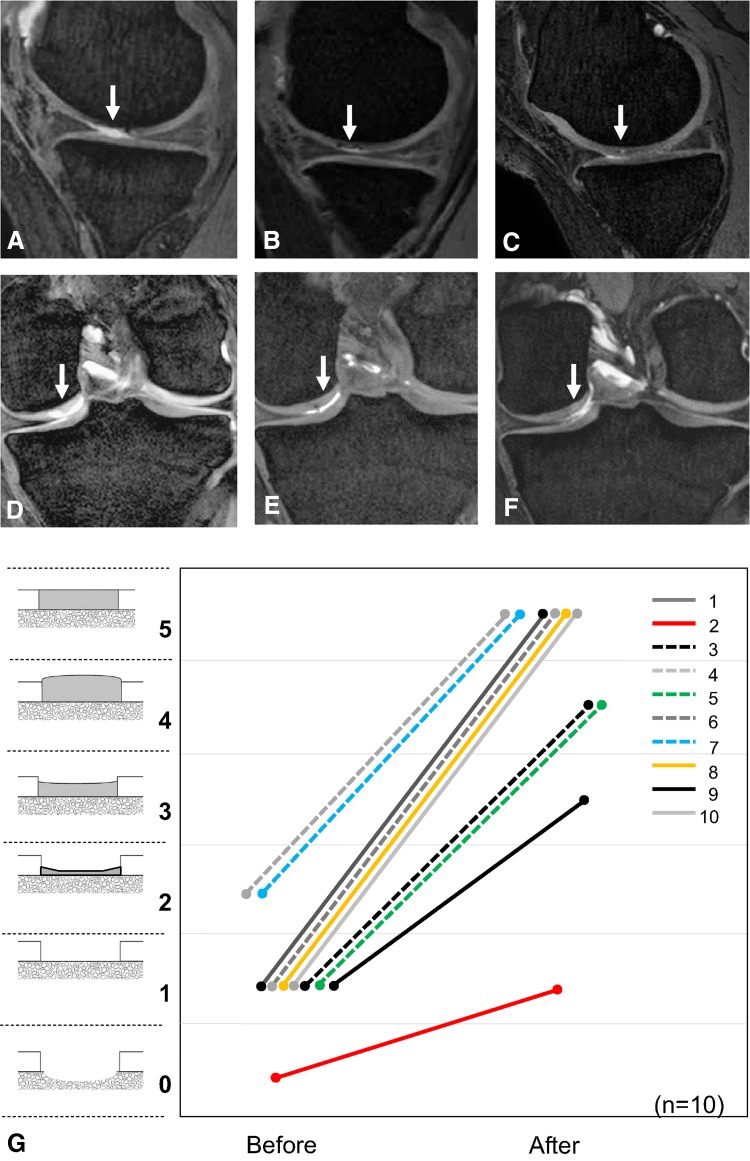
Sequential MRI features and the MRI scores for the cartilage defects are shown. The cartilage lesion in Patient 8 is indicated by the arrow (**A**) before treatment (**B**) at 6 months, and (**C**) at 2 years. In Patient 10 the cartilage lesion is indicated by the arrow (**D**) before treatment, (**E**) at 3 months, and (**F**) at 2 years. (**G**) The MRI scores for the cartilage defects are shown (n = 10 patients; p = 0.005 by Wilcoxon signed rank test between MRI scores before and after transplantation of synovial MSCs). “Bone defect” is scored as 0; “Subchondral bone exposure” is scored as 1; “Cartilage defect extending down more than 50% of cartilage depth” is scored as 2; “Cartilage defect extending down to less than 50% of cartilage depth” is scored as 3; “Cartilage hypertrophy” is scored as 4; and “Complete healing” is scored as 5. Patient number is indicated by the key in the upper right, and the dotted lines indicate MRI scores in patients with ACL reconstruction.

Based on MRI evaluations, cartilage defects were covered with cartilaginous tissue with time (Fig. 3A–C [Patient 8]). In some instances, cartilage defects already were covered with cartilaginous tissue at 3 months and the repaired cartilage was maintained thereafter (Fig. 3D–F [Patient 10]). MRI scores for cartilage defects increased after treatment for all 10 patients, regardless of ACL reconstruction (Fig. 3G). MRI scores were 1.0 ± 0.3 before and 5.0 ± 0.7 after the treatment (median ± 95% CI, p = 0.005). Lateral femoral condyle defects were incompletely healed in Patient 9, whose lateral meniscus previously had been completely removed.

**Fig. 4A–F:**
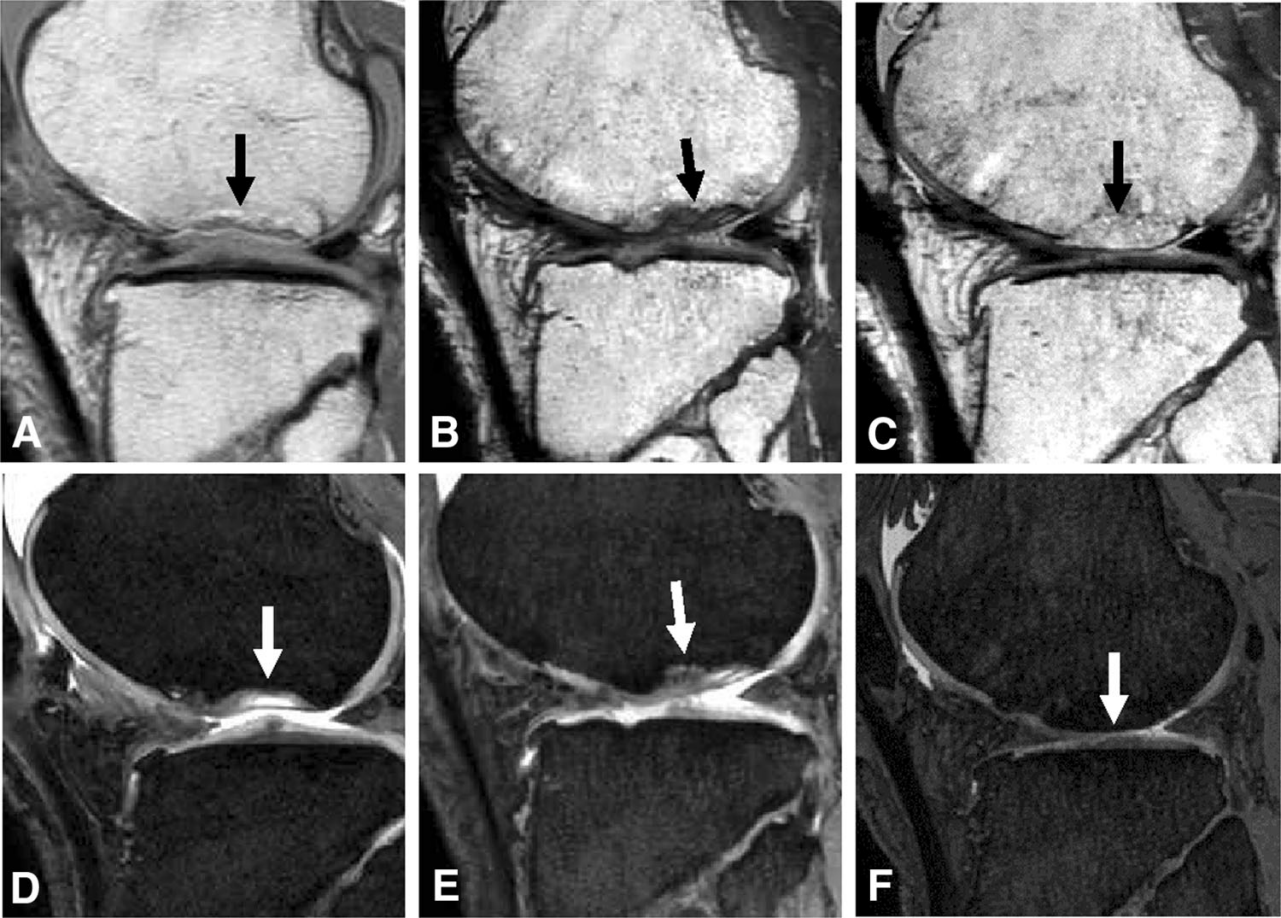
Sequential MRI features of Patient 2 who had an osteochondral defect, indicated by the arrow, are shown (**A**) for bone before treatment, (**B**) at 6 months, and (**C**) at 6 years, and for (**D**) cartilage before treatment, (**E**) at 6 months, and (**F**) at 6 years. The sequential MR images showed that the osteochondral defect was filled with cartilaginous tissue at 6 months. The bone defect was filled with bony tissue, and the cartilage defect was incompletely filled with cartilaginous tissue at 6 years.

Patient 2 presented with an osteochondral defect of the lateral femoral condyle and reported severe pain in the knee before treatment. At 72 months, the bone defect already was filled with bone-like tissue (Fig. 4A–C), and the cartilage defect was incompletely filled with cartilaginous tissue (Fig. 4D–F). The patient’s symptoms gradually improved after transplantation of synovial MSCs.

**Fig. 5A–L:**
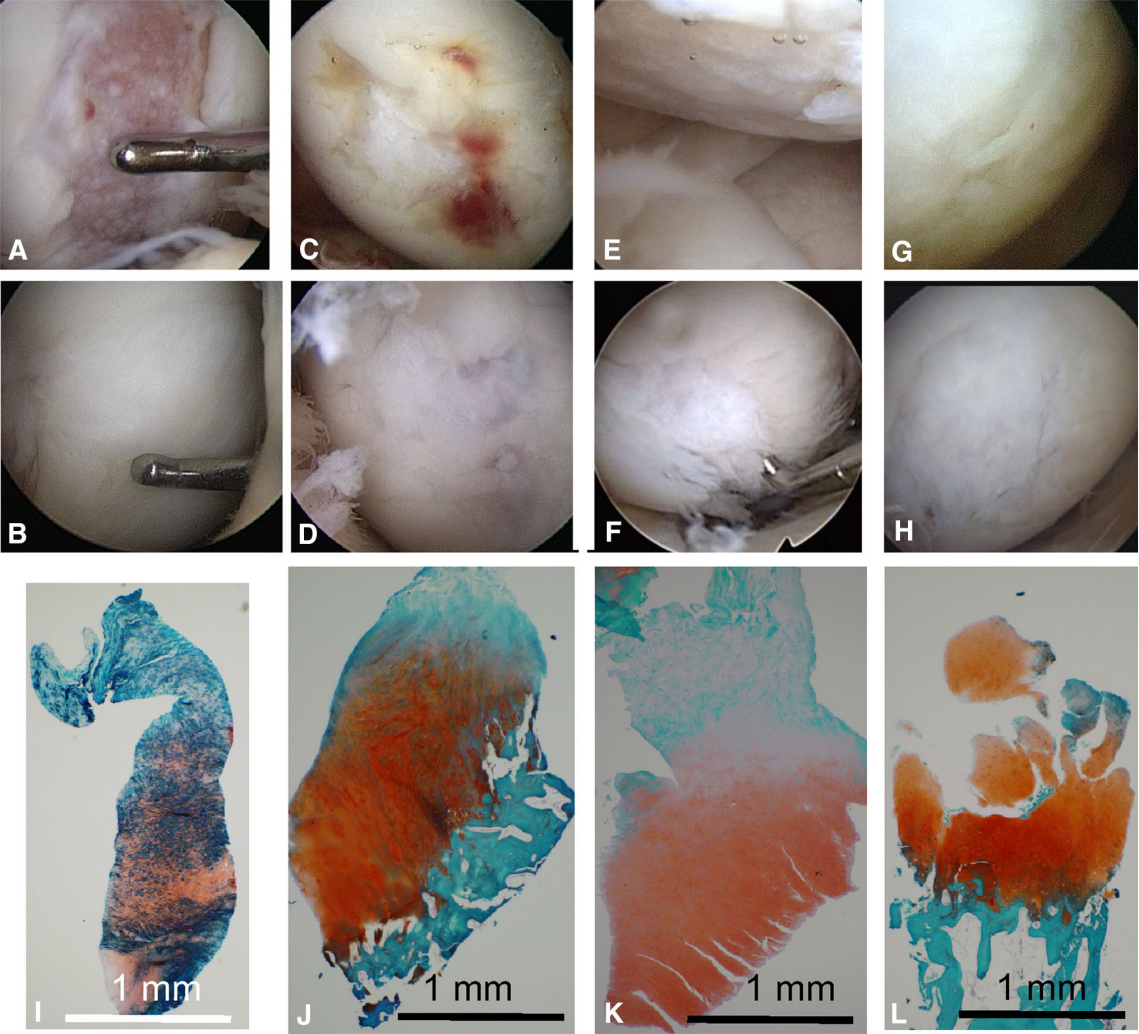
Arthroscopic and histologic assessments were performed before and after transplantation of synovial MSCs. Arthroscopic features for the cartilage in Patient 3 are shown (**A**) before treatment and (**B**) at 11 months; for Patient 4 (**C**) before treatment and (**D**) at 18 months; for Patient 5 (**E**) before treatment and (**F**) at 14 months; and for Patient 7 (**G**) before treatment and (**H**) at 12 months. (**I**) A histologic section of the repaired cartilage obtained after a needle biopsy at the center of the repaired cartilage in Patient 3 contained fibrous cartilage in the deep zone and fibrous tissue in the surface zone. (**J**) In Patient 4, the histologic section contained hyaline cartilage in the deep zone and fibrous tissue in the surface zone. (**K**) In Patient 5, the section contained hyaline cartilage in the deep zone and fibrous tissue in the surface zone, and (**L**) in Patient 7, the section contained hyaline cartilage in the deep zone (Stain, Safranin O and fast green; Bar = 1 mm).

The repaired cartilage was examined arthroscopically and histologically for the four patients who had staples for ACL reconstruction removed (Fig. 5). The cartilage defect appeared improved in all four (Fig. 5A–H). In Patient 3, the surface of the repaired cartilage appeared hypertrophic but was soft, although its deep zone was cartilaginous (Fig. 5A, B); therefore, an additional procedure was not performed. In Patient 4, the thickness of the cartilage defect appeared to increase at 18 months (Fig. 5C, D). In Patient 5, the surface of the repaired cartilage consisted of fibrillated fibrous tissue (Fig. 5E, F), which was removed because the patient reported a minor catching sensation before the second-look arthroscopy. The symptom disappeared after the additional procedure. In Patient 7, the thickness of the cartilage defect also appeared to increase at 12 months (Fig. 5G, H).

Specimens from Patient 3 contained fibrous cartilage in the deep zone and fibrous tissue in the surface zone (Fig. 5I). Specimens from Patients 4, 5, and 7 contained hyaline cartilage in the deep zone (Fig. 5J–L). However, specimens from Patients 4 and 5 consisted of fibrous tissue in the surface zone (Fig. 5J, K).

Lysholm knee scores improved after treatment in all 10 patients regardless of ACL reconstruction (Table 3). The Lysholm score was 76 ± 7 before and 95 ± 3 after the treatment (median ± 95% CI, p = 0.005). The Tegner Activity Scale score did not decrease after the treatment in all 10 patients (Table 3). There were no complications observed up to 37 months at minimal followup, except in Patient 5 who had fibrillation of the repaired cartilage.

**Table 3:** Outcomes before and after transplantation of synovial MSCs

Patient	Lysholm score		Tegner activity scale		Activity
	Before*	After*	Before	After	
1	76	81	7	7	Recreational soccer
2	48	95	5	5	Heavy labor
3	62	95	4	4	Cycling
4	80	95	7	7	Recreational basketball
5	73	95	7	7	Recreational basketball
6	76	94	6	6	Recreational volleyball
7	76	90	6	6	Physical education
8	75	100	6	6	Recreational tennis
9	86	95	6	6	Recreational tennis
10	86	100	3	3	Light labor

* Lysholm scores before and after transplantation were 76 ± 7 and 95 ± 3 (median ± 95% CI, p = 0.005 by Wilcoxon signed-rank test).

## Discussion

MSCs provide promising candidate cells for therapy. In this clinical study, we examined whether transplantation of synovial MSCs improved MRI features, histologic features, and clinical evaluation scores in patients with cartilage defects in the knee. For this small, initial case series, transplantation of synovial MSCs was effective in terms of MRI, qualitative histologic findings, and Lysholm score.

Our study has four limitations. First, the study included only 10 patients who presented with various preoperative conditions. Five patients had ACL reconstructions, among whom two had medial meniscus sutures performed simultaneously. The associated surgeries may have affected the outcome of the transplantation. Second, the number of patients for whom second-look arthroscopy and biopsy results were available also was limited. Third, we observed promising outcomes for use of synovial MSC transplantation, but longer-term observations will be necessary to fully evaluate this new treatment. Fourth, we have not performed prospective comparative randomized trials among synovial MSCs, mosaicplasty, marrow stimulation, and chondrocyte transplantation approaches and we did not use validated patient-reported outcomes tools such as the Knee Injury and Osteoarthritis Outcome Score [20]. These comparisons and measures will be essential to show the effectiveness of synovial MSC transplantation for routine clinical use. Finally, while not strictly a limitation, this study included one patient who previously had a total meniscectomy but who did not receive a simultaneous meniscus replacement (scaffold or allograft). This currently is considered an exclusion criterion for autologous chondrocyte implantation, together with malalignment and laxity [2]. We started this clinical study in 2008. At that time, excluding a patient with a total meniscectomy, to our knowledge, was not the standard course therefore the patient was included here.

For repair of cartilage, bone marrow stimulation, mosaicplasty, and autologous chondrocyte implantation are currently the most commonly performed procedures [14]. Bone marrow stimulation techniques such as microfracture still represent a simple first-line option when performed in young patients with small, single lesions, and low postoperative demands; however, larger lesions and active patients require an alternative procedure [13]. Mosaicplasty and autologous chondrocyte implantation usually require open surgery and the sacrifice of normal, healthy cartilage tissue. The development of cartilage repair procedures that are effective but minimally invasive is ongoing.

Bone marrow is currently the most common MSC source used clinically. Wakitani et al. [30] performed a prospective clinical study of bone marrow MSC transplantation for cartilage repair in which passaged bone marrow MSCs were resuspended in a collagen type I gel and transplanted with an autologous periosteal flap in patients with medial osteoarthritis in the knee who underwent a high tibial osteotomy. They [31] also presented three case reports where a bone marrow MSC-containing scaffold with a periosteal flap was used. Nejadnik et al. [18] performed an observational cohort study in which the clinical outcomes of patients treated with autologous chondrocyte implantation were compared with outcomes of patients treated with autologous bone marrow MSCs. The patients had the periosteum sutured to the cartilage defect which was sealed with fibrin glue; bone marrow MSCs then were implanted beneath the patch. Transplantation of bone marrow MSCs improved the symptoms of the patients and cartilage lesions [32].

Another possible problem related to the use of bone marrow-derived MSCs for cartilage repair is phenotype stability because of their intrinsic tendency to undergo endochondral ossification and consequently calcify, forming subchondral bone overgrowth or intralesional osteophytes. We did not observe any subchondral bone overgrowth or ectopic bone formation although we analyzed only 10 cases by radiographs and MR images. It currently is not possible to definitively conclude whether bone marrow MSCs or synovial MSCs produce more stable forms of cartilage.

From our study results, we propose three potential advantages to using our procedure with synovial MSCs instead of procedures with bone marrow MSCs. First, we could prepare passage 0 synovial MSCs, expanded with autologous human serum in 14 days, for transplantation. We previously attempted to expand passage 0 synovial MSCs and bone marrow MSCs with autologous human serum. More than 10 million synovial MSCs were obtained from all nine donors, contrary to more than 1 million bone marrow MSCs from only two among nine donors [19]. In the current study, we also were able to prepare more than 30 million synovial MSCs with autologous human serum from nine of 10 patients and confirm no chromosomal abnormality in synovial MSCs in all cases. Passage 0 cells are safer than cells passaged several times in terms of the probability of developing chromosome abnormalities [5]. In addition, the ability to prepare enough passage 0 cells in 14 days could reduce costs compared with the need to passage cells multiple times for longer periods. Second, we could transplant synovial MSCs arthroscopically, allowing patients to return to daily life and sports activities earlier than those with more open surgery. Third, scaffolds were not used in our current procedure, which can reduce possible risks such as foreign body reactions [17] and delay the natural healing process [8]. In the current clinical study, the maximum size of the lesion was 500 mm^2^. For larger defects, some modification may be required.

The repair process for osteochondral defects after transplantation of synovial MSCs is of considerable interest. In a rabbit study, osteochondral defects were first filled with cartilage matrix, then the integrated border region between bone and cartilage progressed upward, and finally, the entire thickness of the regenerated cartilage became similar to that of the neighboring cartilage [7]. A similar repair process was reported with bone marrow MSCs in a rabbit model [28]. By contrast, when chondrocytes were transplanted, the osteochondral defect was filled with cartilage matrix and this was preserved without remodeling [29]. In the current study, Patient 2 had an osteochondral defect. According to MRI examinations, the defect appeared to be filled with cartilaginous tissue at 6 months, then the bone defect was almost completely filled with bony tissue and the surface of the lateral femoral condyle was partially covered with cartilaginous tissue thereafter (Fig. 5). Similar repair processes were observed in the osteochondral defect after transplantation of synovial MSCs in rabbits and humans although it required a longer time in humans than in rabbits.

According to histologic biopsy specimen analyses, fibrous tissue was observed at the surface in three of four specimens. Nakamura et al. [16] reported that placing a synovial MSC suspension on the osteochondral defect for 10 minutes promoted cartilage repair, and sequential arthroscopic observations showed the cartilage defect was first covered with the formation of a membrane before cartilage repair in a pig model. In the current clinical study, 11, 12, 14, and 18 months may be too short for the repaired cartilage to mature after transplantation of synovial MSCs. Even in chondrocyte implantation, 12 months seemed to be too short for the repaired cartilage to mature according to histologic analyses [22].

We found that we could prepare an average of 47 million passage 0 synovial MSCs expanded with autologous human serum. Synovial MSCs could be transplanted arthroscopically without a scaffold. For this small, initial case series, transplantation of synovial MSCs was effective in terms of MRI, arthroscopic or histologic qualitative findings, and Lysholm score. Transplantation of synovial MSCs may be less invasive than mosaicplasty and autologous chondrocyte implantation. The conclusive observation of the effectiveness of this treatment will require comparative studies, especially with more established arthroscopic procedures, such as marrow stimulation techniques.
